# New Appliances and Things Medical

**Published:** 1897-02-13

**Authors:** 


### NEW APPLIANCES AND THINGS MEDICAL.

**[We shall be glad to receive, at our Office, 28 & 29, Southampton Street, Strand, London, W.C., from the manufacturers, specimens of all new preparations and appliances which may be brought out from time to time.]**

### EXTRACT OF MALT AND DEVONSHIRE CREAM.

(Evans, Gadd, and Co., Fore Street, Exeter.)

We have recently received a sample of the above nutritive food. Containing as it does two out of the three essential food elements, it constitutes a particularly useful adjunct to an invalid dietary. Maltine is a peculiarly assimilable carbohydrate, and of all fats the natural cream from cow's milk as it exists in this new preparation is the most easily digested and most readily absorbed from the digestive tract. The malt which is used in the preparation is the well known Exonia brand, and the cream, which is not of the clotted variety, is mechanically separated from Devonshire milk. Malt and cream blended in the proportions selected by Messrs. Evans and Gadd constitute a most palatable preparation; children as well as adults appear to prefer it to either of the simple constituents. Theoretically as a fattener there can be no better combination than malt extract and cream, and such practical tests as we have been applying on a small scale distinctly go to prove the correctness of the assumption. It is certainly a very agreeable substitute for cod liver oil, besides having the additional merit of containing a carbohydrate as well as a fat.

### SARINA AND SARINA TISSUE.

(William Jowett, Cataract Mills, Mellor.)

This is a new surgical dressing with several commendable qualities; it is composed chiefly of a soft vegetable tissue which has powers of absorption considerably greater than ordinary cotton wool; pus and other thick discharges are said to be readily taken up by the tissue and to be distributed throughout its substance instead of clotting and remaining at the point of contact as is frequently the case with ordinary dressings. The tissue as now provided by the firm is sent out as a form of splint padding—that is to say, layers of tissue between layers of gauze, a tidy and convenient arrangement. In cases where great antisepsis is necessary the tissue may be previously impregnated with Izal, and in this form it may be obtained from the manufacturers. In no department of surgery has the progress during recent years been more pronounced than in the form and variety of the dressings; year by year they become more efficient and handy, and this new form of tissue dressing is certainly one of the most useful of the recent advances.

